# Physical and Thermal Characterizations of Newly Synthesized Liquid Crystals Based on Benzotrifluoride Moiety

**DOI:** 10.3390/ma16124304

**Published:** 2023-06-10

**Authors:** Fowzia S. Alamro, Hoda A. Ahmed, Mohamed A. El-Atawy, Muna S. Khushaim, Noha S. Bedowr, Rawan AL-Faze, Nada S. Al-Kadhi

**Affiliations:** 1 Department of Chemistry, College of Science, Princess Nourah bint Abdulrahman University, P.O. Box 84428, Riyadh 11671, Saudi Arabia; fsalamro@pnu.edu.sa (F.S.A.); nsalkadhi@pnu.edu.sa (N.S.A.-K.); 2 Department of Chemistry, Faculty of Science, Cairo University, Cairo 12613, Egypt; 3 Chemistry Department, Faculty of Science, Alexandria University, Ibrahemia, P.O. Box 426, Alexandria 21321, Egypt; 4 Chemistry Department, Faculty of Science, Taibah University, Yanbu 46423, Saudi Arabia; nbedowr@taibahu.edu.sa; 5 Department of Physics, Faculty of Science, Taibah University, P.O. Box 30002, Al-Madina 41447, Saudi Arabia; mkhushaim@taibahu.edu.sa; 6 Strategic Research Labs, Taibah University, P.O. Box 30002, Al-Madina 41447, Saudi Arabia; 7 Department of Chemistry, Faculty of Science, Taibah University, Al-Madinah Al-Munawarah 30002, Saudi Arabia; rafaze@taibahu.edu.sa

**Keywords:** liquid crystals, benzotrifluoride, physical characterizations, DFT, dimorphic

## Abstract

The mesomorphic stability and optical activity of new group-based benzotrifluoride liquid crystals, (E)-4-(((4-(trifluoromethyl) phenyl) imino) methyl) phenyl 4-(alkyloxy)benzoate, or **In**, were investigated. The end of the molecules connected to the benzotrifluoride moiety and the end of the phenylazo benzoate moiety have terminal alkoxy groups which can range in chain length from 6 to 12 carbons. The synthesized compounds’ molecular structures were verified using FT-IR, ^1^H NMR, mass spectroscopy, and elemental analysis. Mesomorphic characteristics were verified using differential scanning calorimetry (DSC) and a polarized optical microscope (POM). All of the homologous series that have been developed display great thermal stability across a broad temperature range. Density functional theory (DFT) determined the examined compounds’ geometrical and thermal properties. The findings showed that every compound is entirely planar. Additionally, by using the DFT approach, it was possible to link the experimentally found values of the investigated compounds’ investigated compounds’ mesophase thermal stability, mesophase temperature ranges, and mesophase type to the predicted quantum chemical parameters.

## 1. Introduction

Between the solid and liquid phases of soft matter, liquid crystals (LCs) represent a unique subclass [1,2,3]. Liquid crystal is commonly called a mesomorphic state; it has properties that combine those of crystals and liquids, and exhibits distinct electro-optic phenomena not found in either [1]. Recently, mesomorphic materials have garnered a lot of attention due to their very beneficial and fascinating applications in a variety of scientific fields, including Pancharatnam–Berry (PB) microlenses [4], reality (AR) and virtual reality (VR) devices [5], biosensors [6], liquid crystal elastomers [7], organic field-effect transistors (OFETs), and photovoltaics [8]. When an electric field is applied to a material that contains chiral difluorinated side chains, it causes the disruption of nanofilament crystals and the production of a tilted smectogenic phase [9].

Designing and producing novel, low-cost mesomorphic compounds with high thermal stability and a broad mesomorphic range will always present with issues [3]. However, one of the most effective ways to produce novel, inexpensive liquid crystal materials with good characteristics is through chemically altering the structure [3]. A new low-cost LC material with devolved properties that is suitable for display technology can be created by making even relatively small changes to the molecule’s molecular architecture, such as adding heteroatoms or different lateral moieties [10]. These changes can have a significant impact on the molecule’s mesomorphic interactions, structural geometry, transition temperature, preferred conformations, and other fundamental physical characteristics [10].

A typical liquid crystal molecule consists of two major components: a mesogen, the center rigid part, and a spacer representing the flexible terminal alkyl chains. Additionally, the calamitic liquid crystal molecule includes a central linkage part that connects the two (or more) ring systems [1,11]. For systems with a Schiff’s base/ester group, Schiff base (–C=N–) is a connecting group connecting the hardcore LCs, and, as recently discovered, the twist-bend smectic mesophases [12,13]. The rigid linear Schiff base bridges maintains the linearity of the structure’s core, thereby allowing for the formation of LC phases with extreme stability [14,15].

For the terminal part, it has been demonstrated that the terminal substituents can be alkoxy chains or small polar compact substituents [16,17]. The terminal alkoxy chain of a liquid crystalline compound, in general, has a significant impacts in the development, thermal stability, type, and stability range of the mesophases of liquid crystal [13,18,19,20,21,22,23,24]. According to the investigation findings [17], an increment in the terminal substituent length results in the molecules being oriented in a parallel arrangement, thereby promoting the formation of the smectic A phase. However, polar substituents such as the trifluoromethyl group increase molecules’ polarity, leading to higher dielectric anisotropy, which is important for electro-optical applications. [17,25]. The increased dipole moment enhances both the melting point and the lattice stability of the material [17,26]. Moreover, the introduction of the trifluoromethyl group, which is highly stable and inert, is useful in the design of liquid crystalline materials with improved thermal stability and resistance to degradation. In addition, their incorporation into a liquid crystalline molecule can result in changes in the mesogenic properties, such as the type and strength of the intermolecular interactions and mesophase stability. The trifluoromethyl group can increase the solubility of a liquid crystalline molecule in organic solvents, which is important for the synthesis and processing of these materials.

In recent years, it has been observed that the mesomeric interactions of Schiff bases/ester systems are significantly influenced by the terminal polar substituent, especially the F atom [27]. The location and spatial orientation of the F atom significantly impact this result. The melting and mesomorphic transition temperatures, the dipole moment, the morphology of the mesophase, and the dielectric anisotropy of the resulting mesomorphic compound were all significantly affected by the minimal volume and high polarity of the lateral F atom. [28,29,30,31,32]. Thus, suitable mesogenic moieties and terminal group selection are critical factors in creating novel thermotropic LCs with new phase transitions [17].

Several researchers [12,14,17,33,34,35,36,37,38,39] have demonstrated that computational studies are a great tool for designing novel materials. Molecular orbital energies, the frontier molecular orbital energy difference, and the molecular geometries of the investigated LC compounds must all be stimulated to produce novel compounds with appropriate optical and thermal properties. Due to its high performance and accurate results, density functional theory (DFT) is widely acknowledged as an appropriate instrument for making these predictions [39]. In addition, one of our research areas focuses on theoretical calculations of molecular geometry that impact thermal properties and links them to experimental data [12,33,40,41].

**Figure 1:**
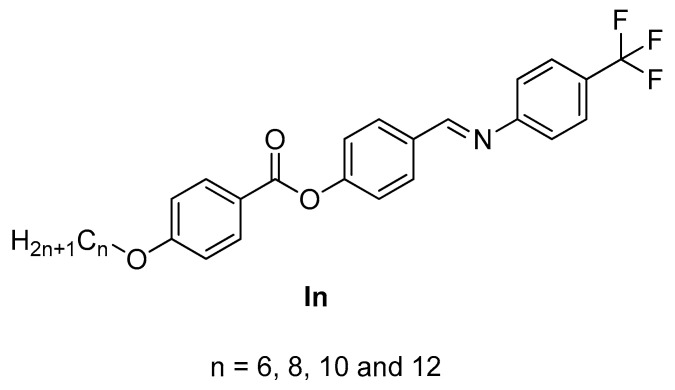
The structure of the liquid crystals, (E)-4-(((4-(trifluoromethyl) phenyl) imino) methyl) phenyl 4-(alkyloxy)benzoate, or **In**.

Based on the above formation, the study aims to prepare a new Schiff base with ester-derived liquid crystals and investigate the terminal benzotrifluoride moiety’s influence on the resulting derivatives’ phase behaviors. Therefore, a series of LCs (**In**), namely, (E)-4-(((4-(trifluoromethyl) phenyl) imino) methyl) phenyl 4-(alkyloxy)benzoate, (Figure 1), were synthesized. The length of the terminal carbon chain attached to an end, where hexyloxy, octyloxy, decyloxy, and dodecyloxy chains are utilized, distinguishes the compounds in the series from one another. DSC and POM instruments were used in the LC study for the novel series to characterize the thermal and mesomorphic behavior. DFT will be used as a theoretical structure to examine these findings. Additionally, these computations show how the terminal moieties influence mesomorphic properties.

## 2. Results and Discussion

### 2.1. Liquid Crystal Study

**Figure 2:**
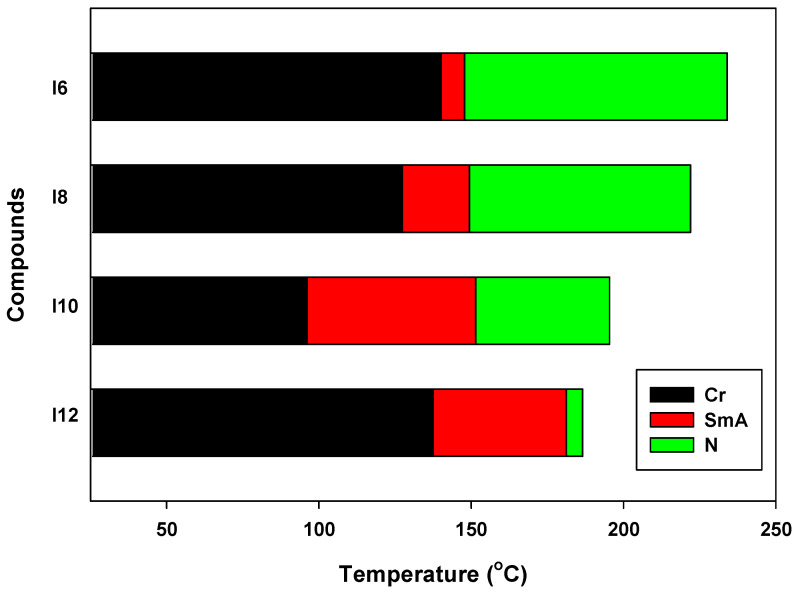
The prepared materials’ phase behavior was observed after the second heating for the **In** series.

**Figure 3:**
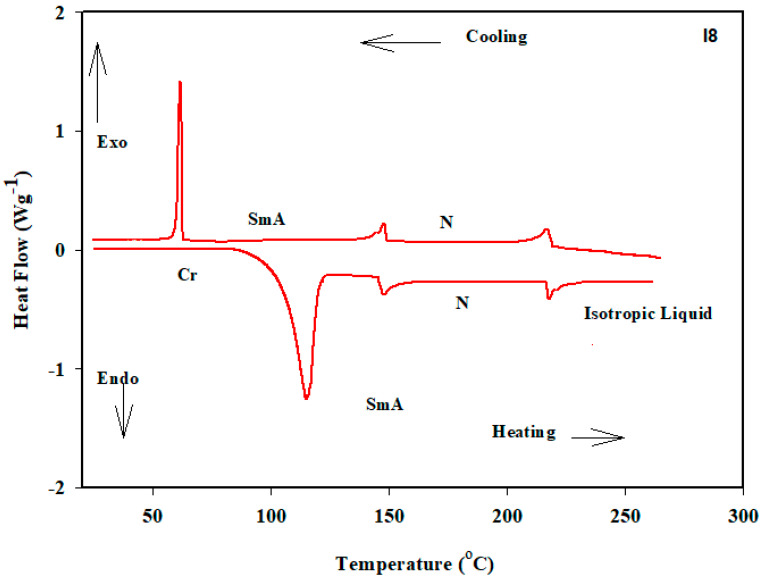
DSC thermograms of **I8** were recorded from the second heating scan (endothermic process) and from the second cooling scan (exothermic process) with a rate of 10 °C/min (red line).

The transition temperatures and corresponding enthalpies for each synthetic material from the DSC experiments are shown in Table 1 and visually represented in Figure 2. All derivatives were thermally stable, as demonstrated by the consistency of the heating and cooling from DSC curves. Figure 3 shows the heating and cooling scans from the DSC for substance **I8**. The second heating scan measures the transition temperatures and enthalpy estimations.

**Figure 4:**
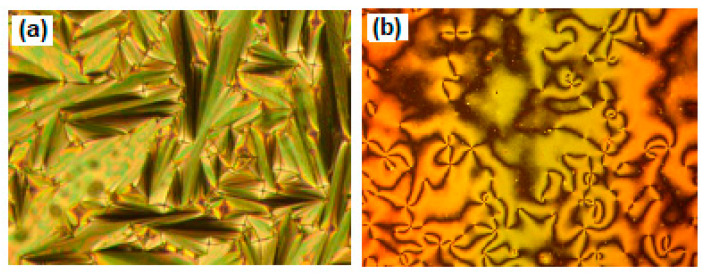
POM textures of **l8** compound during heating: (**a**) SmA phase at 140 °C and (**b**) N phase at 180 °C.

After heating, the DSC thermogram of the formed compound (**I8**, shown in Figure 2) revealed three endotherms typical of the Cr-SmA, SmA-N, and N-I transitions during both heating and cooling scans. While the compound displays the nematic and SmA phases during the cooling cycle, their transitions are delayed to slightly lower temperatures than during the heating cycle. SmA and N mesophases have been verified by textures seen in the POM measurements (Figure 4). While N had a threads/Schlieren-type texture, the SmA phase displayed a focal conic fan texture. This demonstrated that the substance had enantiotropic dimorphic characteristics. Figure 2 depicts the graphic transition temperatures of all the produced materials evaluated so one can assess how the length of the terminal carbon chain impacts how they behave in the mesophase.

All synthesized compounds contain mesomorphic phases, as indicated in Table 1. Additionally, each one exhibits the same types of mesophases with LC phases across a range of temperatures, with the thermal stability changing with the length of the terminal alkoxy carbon chain (an example of **I8** in Figure 3). Additionally, under crossed polarizers, typical images for SmA and N phases (Figure 4) produced by conventional linear LCs could be seen. Data from Table 1 and Figure 2 shows that the terminal chain length increased from n = 6 to 12, and the examined derivatives’ melting temperatures (Cr-N/SmA) exhibited a different pattern. As the polarizability of the investigated derivatives in the same series rises, the melting point rises. The observed present trend, however, did not follow this general principle. All of the homologous series’ members are also enantiotropic, have a broad temperature mesomorphic range as well as excellent mesophase thermal stability. The compounds of the current group (**In**) have enantiotropic SmA and N phases and are dimorphic. The SmA range (*Δ*T_SmA_ = T_SmA_ − T_cr_) is increased from 7.7 to 55.4 °C with an increasing alkoxy chain length from n = 6 to 10, then decreases to 43.7 °C at n = 12, while the N phase range (*Δ*T_N_ = T_iso_ − T_SmA_) decreased from 86.2 to 5.3 °C as the alkoxy chain length n minimized from 6 to 12. As the terminal chain length increased, the development of the SmA phase decreased the nematic phase range. This was most likely caused by an increase in the van der Waals interactions between the lengthy alkoxy chains, which caused them to intertwine and made it easier for the lamellar packing—which is essential for the development of the smectic phase—to occur. However, the mesophase range is significantly influenced by the molecule’s structural shape.

For all compounds with lengthening terminal alkoxy chains, the nematic phase’s stability falls while the SmA phase normally rises [42,43]. The mesogenic rigid core dilution is responsible for the downward trend in the thermal transition of the N phase. Nevertheless, as the alkoxy chain length increased, the SmA phase appeared and lowered the nematic phase range. This is most likely caused by an increase in the van der Waals interactions between long alkoxy side chains, which leads to their interweaving and makes lamellar packing—essential for the emergence of the smectic phase—more convenient. The stability of the generated mesophases and their images is generally attributed to the polarity of the substituent groups, polarizability, aspect ratio, stiffness, and geometry of the molecule. These elements influence the behavior of the mesophase to varying degrees. The stability of a mesophase of a specific mesomorphic compound is known to increase with any improvement in the polarity and/or polarizability of the mesogenic core of the molecule, which is influenced by the polarity of the substituent and subsequently affects the polarity of the entire molecular structure.

Additionally, by favoring lamellar packing, increment van der Waals interactions, which grow with the increase in terminal alkoxy groups, improve the stability of the SmA phase. The nematic phase range, however, was suppressed. The next section will discuss these factors, their types, how they relate to the parameters of quantum chemistry and molecular geometry explored by DFT calculations, and how they influence the stability of mesophases.

For the examined compounds, the estimated entropy change of mesophase transitions (***∆S***/R) is shown in Table 2. Small magnitudes of the ***∆S***/R associated with the SmA-N and N-isotropic entropy are observed, along with an irregular trend independent of the molecules’ (n) terminal alkoxy chain length. However, the tiny values in all derivatives may be caused by the ester linkage group’s slight induction of molecular biaxiality and the comparatively high clearing temperature values, limiting SmA-N and N-isotropic entropy changes [44,45,46]. However, modifications to the interactions between molecules, which are governed by their geometrical shape, aspect ratio (length/breadth ratio), polarizability, stiffness, and dipole moment, may be used to explain the variance and complexity in the entropy change. These elements may have varying degrees of impact on the translational, orientational, and conformational entropies of the molecule, although an increase in alkoxy chain length dilutes interactions between cores and improves the polarizability of the overall molecule, which increases the strength of intermolecular adhesion interactions between nearby molecules and encourages a greater degree of molecular ordering. Removing the long orientational order and the rise in conformational distributions at mesophase interactions are likely responsible for increased ***∆S***/R values with increasing alkoxy chain carbon number. This was further reinforced by the SmA-N transition’s higher measured transition enthalpy value than that recorded for the N-Iso transition.

**Figure 5:**
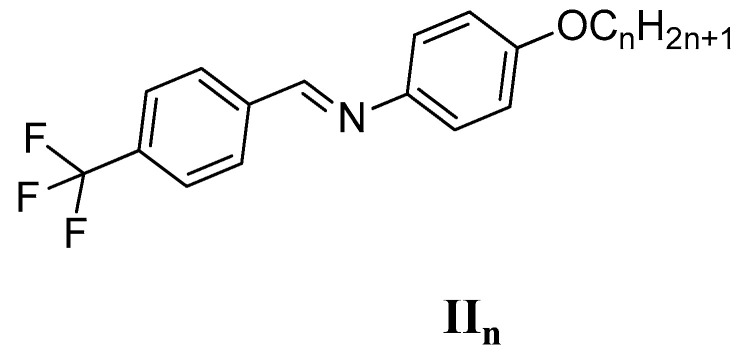
The structure of the liquid crystals previously reported similar to two-ring materials, (**IIn)**.

The comparison of our current series to previously described similar two-ring materials, **IIn** (Figure 5) [47], is interesting. According to the most recent data for **the IIn** series [47], all compounds are mesomorphic and have purely nematic phase types with either enantiotropic or monotropic LC phases, mainly depending on the type of terminal moiety. It can be concluded that the smectic A phases were reported to be generated in the present series **In** by adding extra phenyl ester moiety to the molecular structure of **IIn**, and the resulting phase displayed a wide mesomorphic range and stability. This comparison shows that the current compounds produce more orderly mesophases because their mesogenic parts are longer, typically stabilizing the observed phases.

### 2.2. DFT Studies

**Figure 6:**
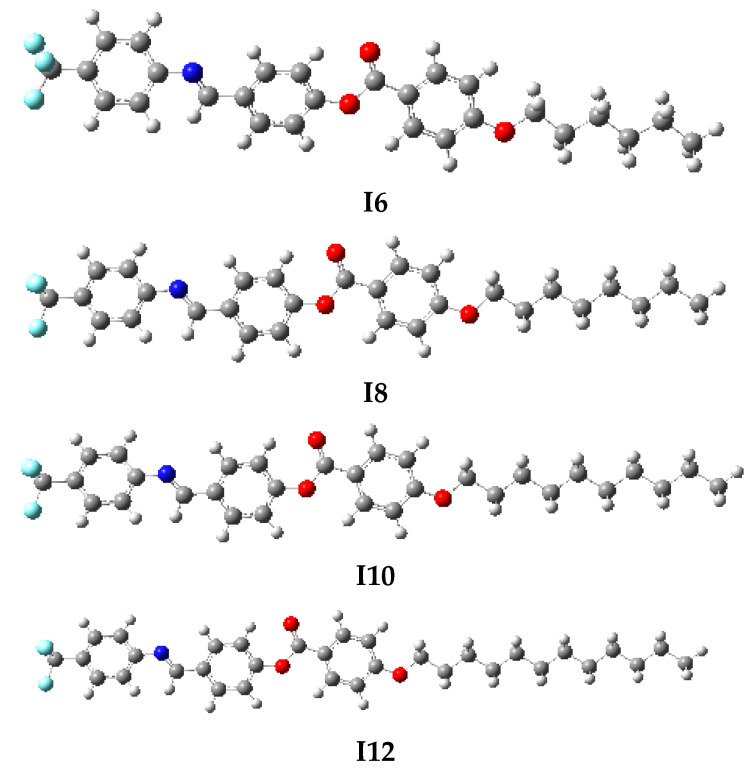
Optimized geometries of the compounds **I6, I8, I10** and **I12** at the B3LYP/6-31+G(d,p) level of theory.

The homologous series **In**’s optimized molecular structures are shown in Figure 6. The energetics and thermodynamic characteristics are listed in Table 3.

The co-planarity of the liquid crystals is a critical and vital factor in determining mesophase behavior. Furthermore, in the liquid crystalline condensed phase, the degree of packing of molecules is highly dependent on their ability to maintain their planar shape. Figure 6 shows that all structures are almost planar, but the orientation of the two terminal aryl rings with respect to the central benzene ring is out of the molecular plane by around 4.0° and 0.7°. Such small dihedral angle values suggest an insignificant deviation from the planarity of the whole molecule. Moreover, the results show that the molecular geometry, especially the planarity of molecules, does not change considerably upon extending the terminal alkyloxy chain length. As a result, the molecular planarity of homologous series **In** enhances and boosts the degree to which molecules are packed together in the condensed mesomorphic phase.

It is worth noting that our outcomes afford a reasonable expectation of the gas phase’s preferred molecular structure; nevertheless, there is a possibility that the presence of these substances in liquid crystalline condensed phases will result in certain deviations.

The dipole moments and molecular polarizabilities are important in defining liquid crystalline solids because they serve as the foundation for comprehending and predicting intermolecular interactions. The dipole moments and molecular polarizability for the homologue series **In** were calculated at the same theory level and displayed in Table 4. The outcomes indicated that as the side alkoxy chain length enhanced, the polarizability ranged from 370.39 to 442.14 Bohr^3^. Lengthened molecules have readily moved electrons, strengthening the dispersion forces, enhancing their polarizability, and resulting in higher boiling and melting temperatures. Small, compact, symmetrical molecules, on the other hand, are less polarizable and have fewer dispersion forces.

**Figure 7:**
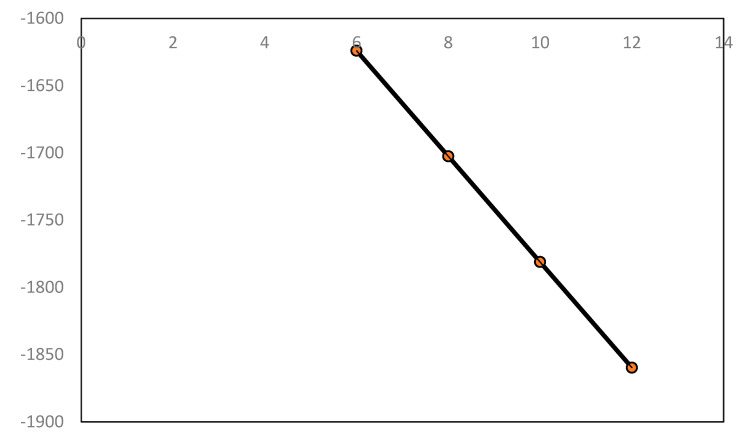
The relationship between the thermal energy of series **In** and the length of the terminal alkoxy chain (●).

To find out the relationship between the stability of the studied molecules and the length of the terminal alkoxy chain, the calculated thermal energy of the homologue series **In** was correlated to the terminal alkoxy chains length (Figure 7). As indicated in Figure 7, a molecule’s stability increases in proportion to the length of the alkoxy chain at its terminal position. The aromatic rings’ high stacking and the aggregation of the alkoxy chains might explain these observations. In terms of benzene ring stacking, the strength of the aggregation rises as the length of the alkoxy chains grows.

Frontier molecular orbitals (FMO) are essential for defining basic molecular features such as stability and optical and electronic properties because they provide a good qualitative estimation of excitation characteristics and electron transport capabilities. The energy levels of the highest occupied molecular orbital (HOMO) and the lowest unoccupied molecular orbital are electron-donating and electron-accepting levels, respectively. The HOMO-LUMO energy gap (*Δ*E) is critical for molecular systems’ nonlinear optical characteristic systems. A low energy gap value is typical of less stable but easily polarizable materials, suggesting an easier electronic transition and, therefore, the greater NLO characteristics of a molecule. A large *Δ*E value is associated with insulation characteristics and strong molecule stability.

In the case of the homologue series **In**, the calculations revealed that the length of the terminal alkoxy chain does not affect the energy values of the FMO. As a result, the length of the terminal alkoxy chain does not influence the energy gap (*Δ*E), and it was found to have the same value for all of the studied molecules. Furthermore, chemical potentials, μ, absolute electronegativity, χ, absolute softness, σ, absolute hardness, η, global softness, S, global electrophilicity, ω, and additional electronic charge, *Δ*N_max_, have been calculated from the FMO energies (Table 4) to shed light on the examined compounds’ stability and reactivity. Hardness is one of the most prevalent and important metrics for understanding the behavior and reactivates of molecules and may be regarded a measure of molecular stability. Softness (S) reveals the degree of the polarizability and photoelectric sensitivity of materials. In addition, *Δ*N_max_ estimaes the maximum number of electrons that may be transferred from the molecule during a chemical reaction; soft molecules would have a high *Δ*N_max_ value.

**Figure 8:**
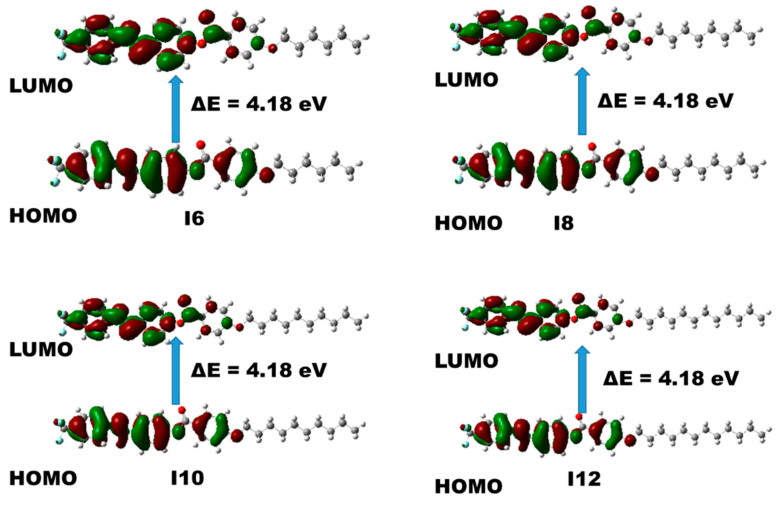
The frontier molecular orbitals for the investigated compounds **In** calculated at the B3LYP/6-31+G(d,p) level of theory.

The calculations revealed that the investigated compounds have a relatively small energy gap *Δ*E (4.18 eV), indicating that the title molecules are soft and reactive. Moreover, as shown in Figure 8, the electron densities of the FMOs are mostly localized on the aryl rings. At the same time, there was no noticeable contribution of the terminal alkyloxy chain to the FMOs. It can be noted that both frontier orbitals have a nearly identical shape in all the investigated molecules.

**Figure 9:**
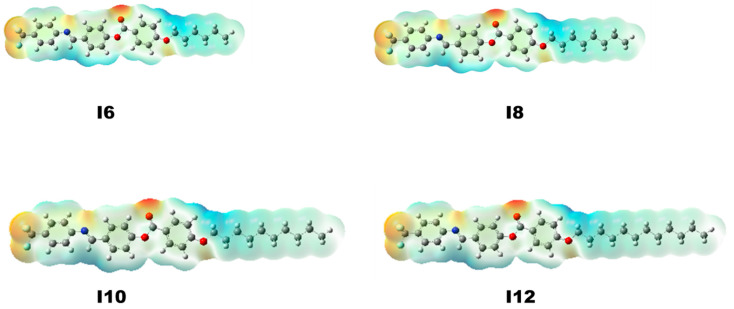
Molecular electrostatic potentials of the homologue series **In**.

The electronic density distribution is shown by the molecular electrostatic potential energy surface (MEP), is a helpful metric that gives some insight on a chemical compound’s electrophilic and nucleophilic centers. Moreover, MEP describes hydrogen-bonding interactions between the molecules. MEP of the **In** series has been calculated utilizing optimal compound geometry at B3LYP/6-31+G(d,p). A greater electron density characterizes the MEP’s negative sites (red) and is regarding with nucleophilic reactivity. Electrophilicity refers to the positive sites, which are either green or blue and have a low electron density. Our potential map’s energy range is −4.576 × 10^−2^ esu to + 4.576 × 10^−2^ esu. In series, as shown in Figure 9, the O, N, and F centers have high electron density and represent the nucleophilic part. In contrast, the low electron density is observed surrounding the alkyl chain.

## 3. Experimental

### Synthesis

**Scheme 1:**
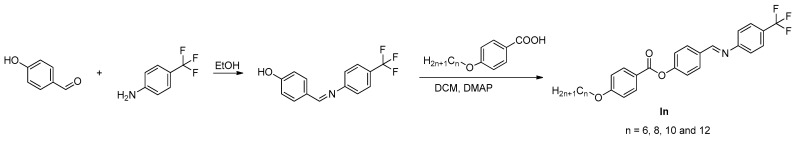
Synthesis of (E)-4-(((4-(trifluoromethyl) phenyl)imino)methyl)phenyl 4-(alkyloxy)benzoate, **In**.

The following Scheme 1 describes the formation of the mesomorphic compounds **In**:

(E)-4-(((4-(trifluoromethyl)phenyl)imino)methyl)phenyl 4-(hexyloxy)benzoate **I6**

Pale yellow crystals yield 92%; m.p. 140 °C. IR (KBr): ῡ 3060 (**sp**^2^ =C–H), 2980 (**sp**^3^ –C–H), 1740 (C=O), and 1610 (C=N) cm^−1^. ^1^H NMR (CDCl_3_, 400 MHz): δ 8.65 (s, 1H, CH=N), 8.11 (d, *J* = 7.7 Hz, 2H, Ar-H), 7.60–7.72 (m, 4H, Ar-H), 7.46 (d, *J* = 7.7 Hz, 2H, Ar-H), 7.14–7.28 (m, 4H, Ar-H), 4.03 (t, *J* = 6.5 Hz, 2H, OCH_2_), 1.56–1.47 (m, 2H, CH_2_), 1.46–1.41 (m, 2H, CH_2_), 1.25–1.20 (m, 4H, 2 CH_2_) and 0.85 (t, 3H, CH_3_) ppm.. C_27_H_26_F_3_NO_3_ (Mwt = 469.19) requires C, 69.07; H, 5.58; N, 2.98 % found: C, 70.12; H, 5.43; N, 3.12 %.

(E)-4-(((4-(trifluoromethyl)phenyl)imino)methyl)phenyl 4-(octyloxy)benzoate **I8**

Pale orange crystals yield 89%; m.p. 127 °C. IR (KBr): ῡ 3043 (**sp**^2^ =C–H), 2966 (**sp**^3^ –C–H), 1741 (C=O), and 1613 (C=N) cm^−1^. ^1^H NMR (CDCl_3_, 400 MHz): δ 8.66 (s, 1H, CH=N), 8.12 (d, *J* = 7.7 Hz, 2H, Ar-H), 7.70–7.61 (m, 4H, Ar-H), 7.44 (d, *J* = 7.7 Hz, 2H, Ar-H), 7.26–7.14 (m, 4H, Ar-H), 4.03 (t, 2H, OCH_2_), 1.75–1.70 (m, 2H, CH_2_), 1.46–1.41 (m, 2H, CH_2_), 1.23–1.20 (m, 8H, 4 CH_2_) and 0.86 (t, 3H, CH_3_) ppm C_29_H_30_F_3_NO_3_ (Mwt = 497.22) requires C, 70.01; H, 6.08; N, 2.82 % found: C, 70.23; H, 5.93; N, 3.02 %.

(E)-4-(((4-(trifluoromethyl)phenyl)imino)methyl)phenyl 4-(decyloxy)benzoate **I10**

Yellow crystals yield 90%; m.p. 96 °C. IR (KBr): ῡ 3060 (**sp**^2^ =C–H), 2950 (**sp**^3^ –C–H), 1742 (C=O), and 1603 (C=N) cm^−1^. ^1^H NMR (CDCl_3_, 400 MHz): δ 8.66 (s, 1H, CH=N), 8.13 (d, *J* = 7.7 Hz, 2H, Ar-H), 7.72–7.61 (m, 4H, Ar-H), 7.44 (d, *J* = 7.7 Hz, 2H, Ar-H), 7.26–7.14 (m, 4H, Ar-H), 4.05 (t, 2H, OCH_2_), 1.78–1.76 (m, 2H, CH_2_), 1.42–1.30 (m, 14H, 7 CH_2_) and 0.88 (t, 3H, CH_3_) ppm C_31_H_34_F_3_NO_3_ (Mwt = 525.25) requires C, 70.84; H, 6.52; N, 2.66 % found: C, 70.63; H, 6.73; N, 2.62 %.

(E)-4-(((4-(trifluoromethyl)phenyl)imino)methyl)phenyl 4-(dodecyloxy)benzoate **I12**

Yellow crystals yield 87%; m.p. 138 °C. IR (KBr): ῡ 3068 (**sp**^2^ =C–H), 2952 (**sp**^3^ –C–H), 1742 (C=O), and 1610 (C=N) cm^−1^. ^1^H NMR (CDCl_3_, 400 MHz): δ 8.66 (s, 1H, CH=N), 8.13 (d, *J* = 7.7 Hz, 2H, Ar-H), 7.72–7.61 (m, 4H, Ar-H), 7.44 (d, *J* = 7.7 Hz, 2H, Ar-H), 7.26–7.14 (m, 4H, Ar-H), 4.06 (t, 2H, OCH_2_), 1.78–1.76 (m, 2H, CH_2_), 1.43–1.31 (m, 18H, 9 CH_2_) and 0.88 (t, 3H, CH_3_) ppm C_33_H_38_F_3_NO_3_ (Mwt = 553.65) requires C, 71.59; H, 6.92; N, 2.53 % found: C, 71.62; H, 6.76; N, 2.61%.

## 4. Computational Methods

See Supplementary Data. 

## 5. Conclusions

(E)-4-(((4-(trifluoromethyl) phenyl) imino) methyl) phenyl 4-(alkyloxy)benzoate, a new three-ring series with –COO and –CH=N connecting units, was synthesized, and the molecular structures were validated by FT-IR, proton and carbon13-NMR, mass spectrometry, and elemental analyses. Studying and correlating the thermotropic and optical behaviors with estimated parameters resulted from the DFT calculations.

The study found that:

1. All produced compounds observe thermal stability with elevated values and wide enantiotropic temperature mesomorphic ranges.
2. The electronic state of the terminal trifluoromethyl moiety and the length of the alkoxy chain cause significant modifications to the intended derivatives’ geometrical parameters.
3. Increasing the aspect ratio of the present series compared to the previously reported two-ring series induces the smectic A phase with broad thermal stability.
4. The findings showed that the estimated polarizability of the present compounds had a similar trend to the influence of aspect ratio on the phase stabilities and their ranges.
5. The electrical characteristics of the terminal groups influenced the global softness and the energy gap of the FMO.

## Figures and Tables

**Table 1:** Mesomorphic transition results for **In**.

Comp.	Mesophase Transition Temperature, °C and Enthalpy of Transitions in kJ/mol	*Δ*T_SmA_	*Δ*T_N_
**I6**	Cr 140.1 (41.7) SmA 147.8 (2.6) N 234.0 (1.4)	7.7	86.2
**I8**	Cr 127.4 (46.6) SmA 149.4 (4.4) N 222.1 (2.0)	22.0	72.6
**I10**	Cr 96.1 (49.2) SmA 151.5 (2.2) N 195.4 (1.5)	55.4	43.9
**I12**	Cr 137.5 (51.9) SmA 181.2 (1.9) N 186.5 (1.2)	43.7	5.3

**Table 2:** Normalized entropy changes * of transitions for formed materials **In**.

Comp.	*∆S* _SmA-N_/R	*∆S*_N-I_/R
**16**	0.74	0.33
**18**	1.25	0.49
**110**	0.62	0.39
**112**	0.50	0.31

* Normalized entropy change (∆S/R) = entropy change/gas constant (dimensionless).

**Table 3:** Parameters for the optimized structures’ energies and thermodynamics at the B3LYP/6-31+G(d,p) level of theory, expressed in Hartree units.

	E_0_	E_298_	H_298_	G_298_	S (cal/mol.kelvin)
**I6**	−1624.007462	−1624.484423	−1623.974637	−1624.078648	218.909
**I8**	−1702.574560	−1703.108607	−1702.539017	−1702.649890	233.351
**I10**	−1781.141646	−1781.732782	−1781.103387	−1781.221127	247.804
**I12**	−1859.708727	−1860.356955	−1859.667753	−1859.792357	262.249

**Table 4:** For the molecules’ optimal configurations for series **In**, various quantum chemical characteristics.

E (a.u)	I6	I8	I10	I12
Dipole moment (D)	9.520	9.570	9.597	9.614
Polarizability (α)	370.39	394.52	418.40	442.14
E_HOMO_ (eV)	−6.528	−6.527	−6.527	−6.527
E_LUMO_ (eV)	−2.348	−2.348	−2.347	−2.347
*Δ*E (eV)	4.180	4.180	4.180	4.180
χ (eV)	4.438	4.437	4.437	4.437
η (eV)	2.090	2.090	2.090	2.090
σ (eV^−1^)	0.478	0.478	0.478	0.479
μ (eV)	−4.438	−4.437	−4.437	−4.437
S (eV^−1^)	0.239	0.239	0.239	0.239
ω (eV)	4.713	4.711	4.710	4.710
*Δ*N_max_	2.124	2.123	2.123	2.123

## Data Availability

The data used for research described in this manuscript are available upon request from corresponding authors: ahoda@sci.cu.edu.eg (H.A.A.); mohamed.elatawi@alexu.edu.eg (M.A.E.-A.)

## Supplementary Materials

The following supporting information can be downloaded at: https://www.mdpi.com/article/10.3390/ma16124304/s1, Figures S1–S3: DCS thermograms of compounds I6, I10 and I12; Figure S4: POM textures of compound I10; Tables S1–S2: Mesomorphic and thermal parameters of compounds In and IIn.
