# Supplementary material for: Physical and Thermal Characterizations of Newly Synthesized Liquid Crystals Based on Benzotrifluoride Moiety
Source: Materials (Basel). 2023 Jun 10;16(12):4304. doi: 10.3390/ma16124304 (PMC10304381; doi:10.3390/ma16124304)
Supplement: Supplementary file 1 [file materials-16-04304-s001.zip › materials-2423802-supplementary.pdf]

Supplementary data

Table S1. Mesomorphic transition results for **In**.

Comp.	transition temperature, °C and (enthalpy of transitions in kJ/mol)	Comp.	transition temperature, °C and (enthalpy of transitions in kJ/mol)
**16**	Cr 140.1 (41.7) SmA 147.8 (2.6) N 234.0 (1.4)	**II6**	Cr 103.2 (42.40) I
**18**	Cr 127.4 (46.6) SmA 149.4 (4.4) N 222.1 (2.0)	**II8**	Cr 97.6 (50.71) N 100.2 (2.37) I
**110**	Cr 96.1 (49.2) SmA 151.5 (2.2) N 195.4 (1.5)	**II16**	Cr 98.9 (53.20) I
**112**	Cr 137.5 (51.9) SmA 181.2 (1.9) N 186.5 (1.2)		

Table S2. Different quantum chemical parameters for the optimized structures of the molecules for series **In and IIn**.

Parameter	I6	I8	I10	I12	II6	II8	II16
E_{HOMO} (eV)	-6.528	-6.527	-6.527	-6.528	-0.21572	-0.21566	-0.21571
E_{LUMO} (eV)	-2.348	-2.348	-2.347	-2.348	-0.07816	-0.07812	-0.07815
ΔE (eV)	4.180	4.180	4.180	4.180	0.13756	0.13754	0.13756
χ (eV)	0.478	0.478	0.478	0.478	0.14694	0.14689	0.14693
μ (eV)	-4.438	-4.437	-4.437	-4.438	-0.14694	-0.14689	-0.14693
η (eV)	0.239	0.239	0.239	0.239	0.06878	0.06877	0.06878
σ (eV^{-1})	4.713	4.711	4.710	4.713	14.53911	14.54122	14.53911
ω (eV)	-6.528	-6.527	-6.527	-6.528	0.15696	0.15688	0.15694

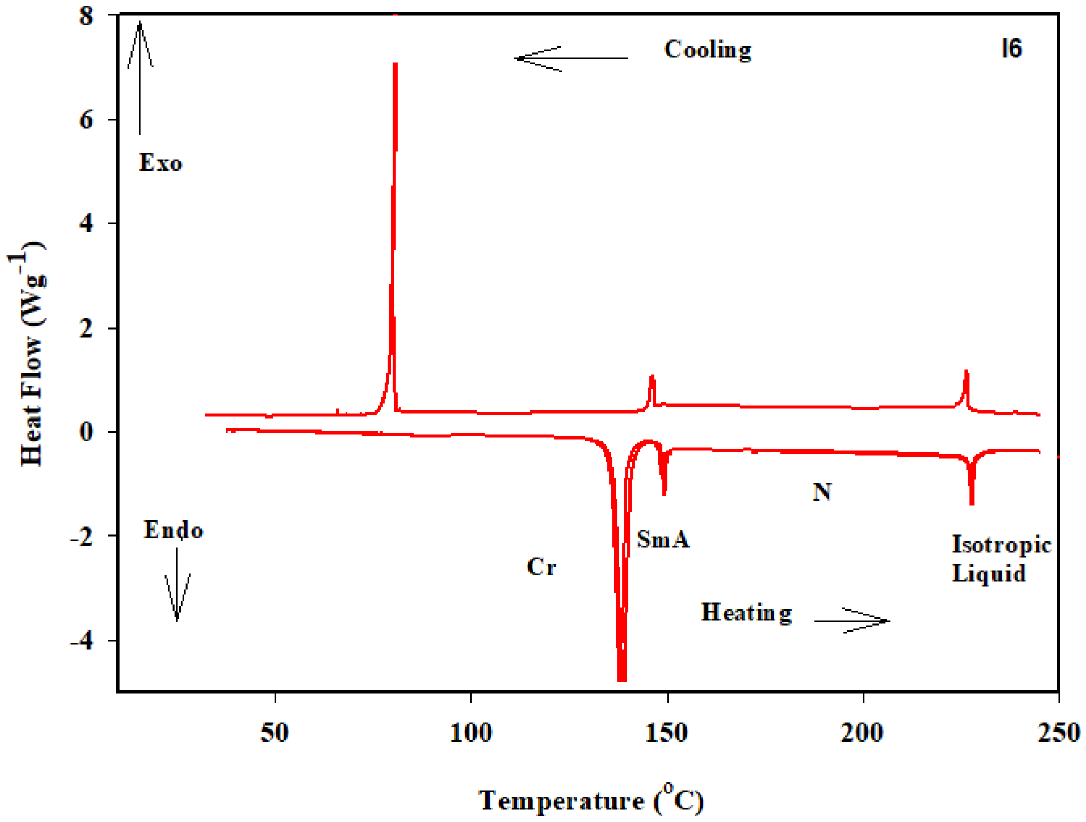

Figure S1. DSC thermograms of **I6**: were recorded from the second heating scan and (b) from the second cooling scan with a rate of 10 °C/min.

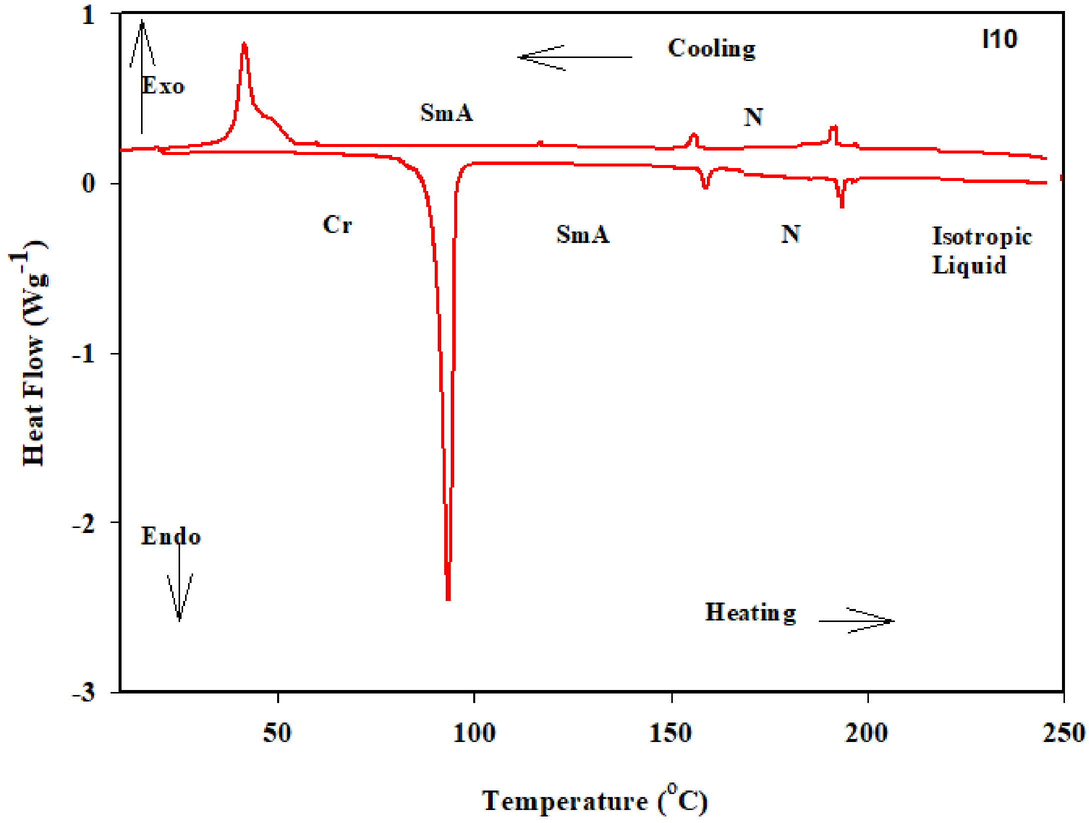

Figure S2. DSC thermograms of **I10**: were recorded from the second heating scan and (b) from the second cooling scan with a rate of 10 ^{o}C/min.

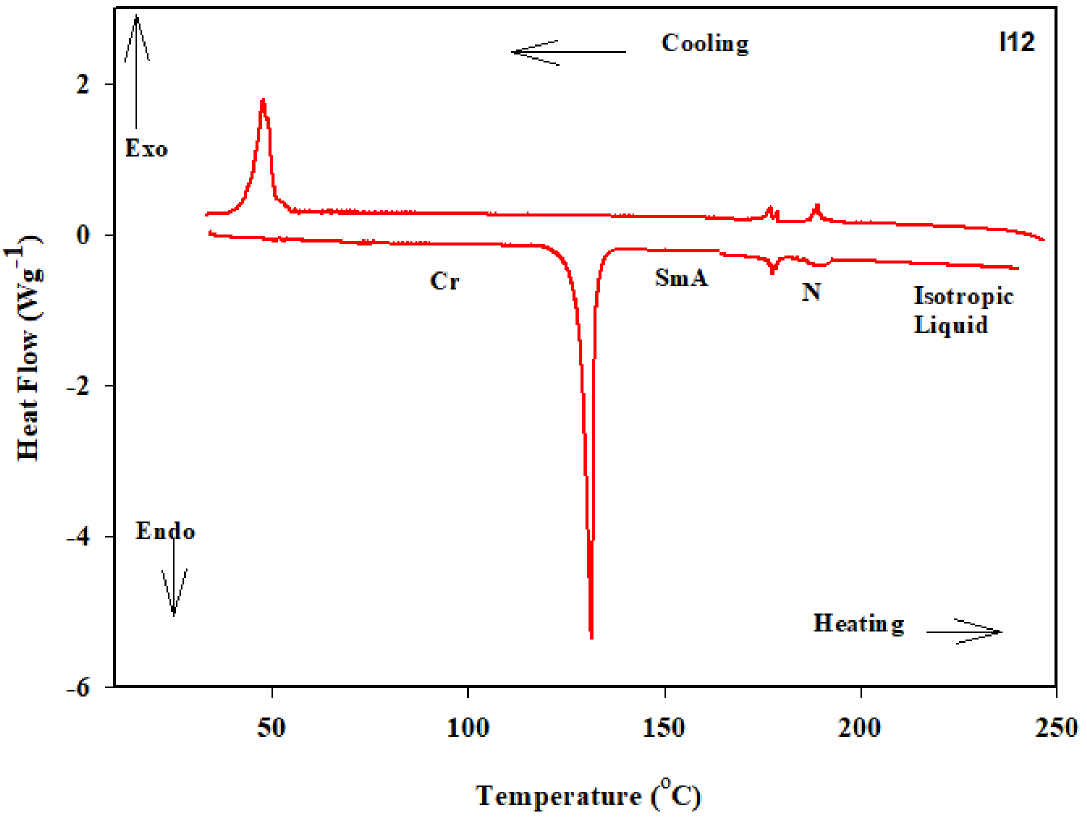

Figure S3. DSC thermograms of **I12**: were recorded from the second heating scan and (b) from the second cooling scan with a rate of 10 °C/min.

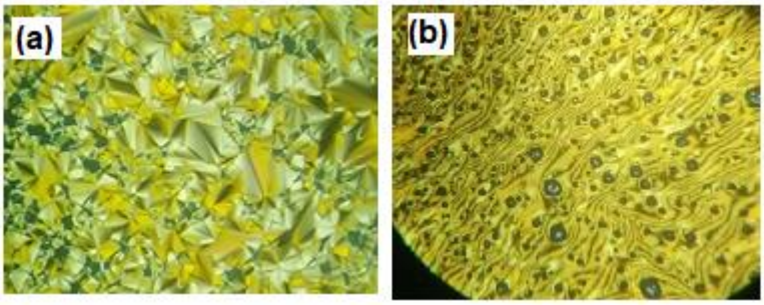

Figure S4. POM textures of **I10** compound during heating ;(a) SmA phase at 120 °C and (b) N phase at 190 ° C.

Optimized geometries (bond length, bend angle and dihedral angle) of the homologous series In at the B3LYP/6-31+G(d,p) level of theory

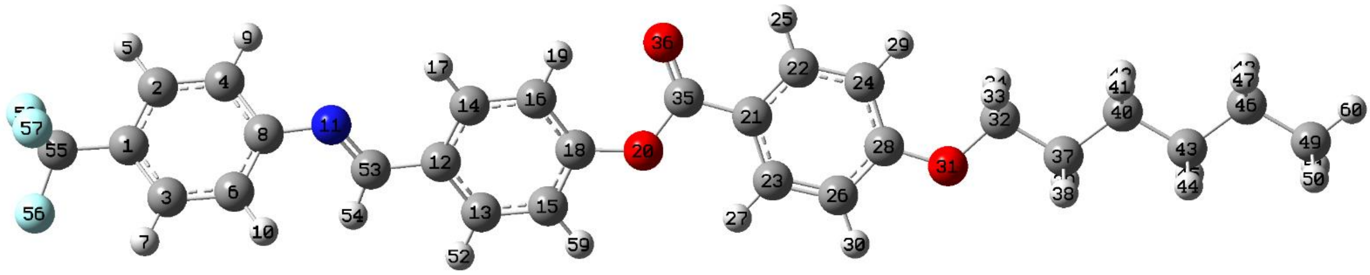

Symbol	NA	NB	NC	Bond	Angle	Dihedral
C						
C	1			1.402193		
C	1	2		1.399189	120.0067	
C	2	1	3	1.388946	119.9596	0.82548
H	2	1	3	1.080183	119.8474	-179.495
C	3	1	2	1.391898	120.0147	-1.46865
H	3	1	2	1.080206	119.8529	179.0295
C	4	2	1	1.406029	120.5038	0.759295
H	4	2	1	1.079901	121.0728	-179.356
H	6	3	1	1.081019	119.8101	178.4824
N	8	4	2	1.409952	117.8693	-179.127
C	11	8	4	2.416778	152.8249	-145.667
C	12	11	8	1.407256	146.3364	8.125332
C	12	11	8	1.406169	94.95044	-172.339
C	13	12	11	1.38979	120.6436	179.5603
C	14	12	11	1.392063	121.3496	-179.74
H	14	12	11	1.080289	118.4762	0.172637
C	15	13	12	1.397706	119.5339	-0.13079
H	16	14	12	1.074857	120.967	179.0934
O	18	15	13	1.410427	113.5541	178.47
C	20	18	15	2.370593	161.3051	168.1023
C	21	20	18	1.403416	151.2684	-2.28349
C	21	20	18	1.409149	89.6357	178.5293
C	22	21	20	1.392131	120.9344	-179.002
H	22	21	20	1.080629	118.7016	0.994744
C	23	21	20	1.385845	120.3257	179.5439
H	23	21	20	1.078613	119.4429	-0.53242
C	24	22	21	1.402611	119.3685	-0.0502

H	24	22	21	1.078987	119.5516	179.9881
H	26	23	21	1.079866	121.6104	179.9604
O	28	24	22	1.382619	124.3193	179.9881
C	31	28	24	1.466321	120.1599	-0.47446
H	32	31	28	1.093863	109.2784	59.80774
H	32	31	28	1.093883	109.2544	-59.2075
C	20	18	15	1.399026	126.1157	167.5931
O	35	20	18	1.234477	123.0582	-0.29572
C	32	31	28	1.520946	107.0093	-179.675
H	37	32	31	1.092775	108.6542	-58.469
H	37	32	31	1.092813	108.5836	57.40705
C	37	32	31	1.53876	112.455	179.4225
H	40	37	32	1.096245	109.5983	58.06716
H	40	37	32	1.096211	109.5484	-58.2233
C	40	37	32	1.538243	112.989	179.9423
H	43	40	37	1.096061	109.2447	-58.141
H	43	40	37	1.096099	109.2343	57.6716
C	43	40	37	1.538221	113.413	179.7573
H	46	43	40	1.095507	109.1645	57.81825
H	46	43	40	1.095474	109.1548	-57.8629
C	46	43	40	1.535857	113.1485	179.9799
H	49	46	43	1.092904	111.1172	-59.8503
H	49	46	43	1.092907	111.1228	59.98573
H	13	12	11	1.082881	119.7795	-0.45005
C	11	8	4	1.289998	122.3997	-141.493
H	53	11	8	1.095895	121.2497	4.610114
C	1	3	6	1.485204	120.0233	-177.077
F	55	1	3	1.401464	112.8153	-29.4458
F	55	1	3	1.40848	112.8808	90.137
F	55	1	3	1.401652	112.7758	-150.431
H	15	13	12	1.079738	121.5278	179.9081
H	49	46	43	1.091793	111.3416	-179.932

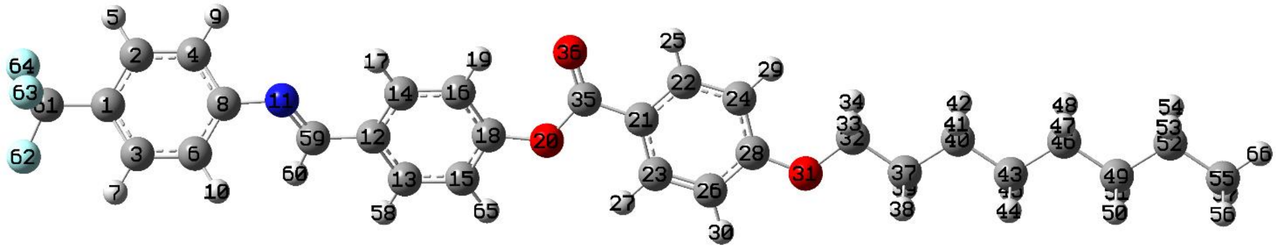

Symbol	NA	NB	NC	Bond	Angle	Dihedral
C						
C	1			1.402197		
C	1	2		1.39919	120.0068	
C	2	1	3	1.38894	119.9597	0.82341
H	2	1	3	1.080184	119.8468	-179.496
C	3	1	2	1.391899	120.0145	-1.46693
H	3	1	2	1.080206	119.8525	179.0308
C	4	2	1	1.406033	120.5037	0.75878
H	4	2	1	1.0799	121.073	-179.354
H	6	3	1	1.08102	119.8104	178.4821
N	8	4	2	1.409939	117.8689	-179.12
C	11	8	4	2.416751	152.8297	-145.679
C	12	11	8	1.407252	146.3399	8.139682
C	12	11	8	1.406177	94.94652	-172.324
C	13	12	11	1.389804	120.6433	179.5601
C	14	12	11	1.392046	121.3487	-179.74
H	14	12	11	1.08029	118.4758	0.172229
C	15	13	12	1.397693	119.5333	-0.13074
H	16	14	12	1.074861	120.9658	179.0823
O	18	15	13	1.410427	113.5613	178.4479
C	20	18	15	2.370645	161.2885	167.9675
C	21	20	18	1.403422	151.2665	-2.34099
C	21	20	18	1.409155	89.63746	178.4797
C	22	21	20	1.392121	120.9351	-178.993
H	22	21	20	1.08063	118.7014	1.002368
C	23	21	20	1.385845	120.3251	179.5408
H	23	21	20	1.078614	119.4436	-0.53624
C	24	22	21	1.402625	119.3681	-0.05236
H	24	22	21	1.078989	119.5538	179.985
H	26	23	21	1.079867	121.6105	179.9596
O	28	24	22	1.382596	124.3178	179.9901
C	31	28	24	1.466353	120.1555	-0.4879
H	32	31	28	1.09386	109.2767	59.8134
H	32	31	28	1.093883	109.2496	-59.1977

C	20	18	15	1.399071	126.1014	167.4285
O	35	20	18	1.234473	123.0537	-0.31295
C	32	31	28	1.520922	107.018	-179.666
H	37	32	31	1.092789	108.6583	-58.4878
H	37	32	31	1.092822	108.5846	57.39112
C	37	32	31	1.538739	112.4469	179.4001
H	40	37	32	1.096225	109.6037	58.06189
H	40	37	32	1.096199	109.5469	-58.2285
C	40	37	32	1.538464	112.9845	179.9428
H	43	40	37	1.095939	109.1992	-58.2099
H	43	40	37	1.095975	109.1834	57.56537
C	43	40	37	1.537975	113.3719	179.6588
H	46	43	40	1.096406	109.2551	57.86637
H	46	43	40	1.096389	109.232	-57.9257
C	46	43	40	1.538037	113.4858	179.9825
H	49	46	43	1.096456	109.2722	-58.0052
H	49	46	43	1.096481	109.2655	57.77668
C	49	46	43	1.538448	113.5383	179.8814
H	52	49	46	1.095546	109.1386	57.81585
H	52	49	46	1.095523	109.1346	-57.8104
C	52	49	46	1.535922	113.2141	-179.997
H	55	52	49	1.092993	111.1066	-59.8429
H	55	52	49	1.092993	111.1105	59.95641
H	13	12	11	1.082881	119.78	-0.45069
C	11	8	4	1.289998	122.4036	-141.499
H	59	11	8	1.095895	121.2498	4.612358
C	1	3	6	1.485202	120.0241	-177.074
F	61	1	3	1.401463	112.8155	-29.4324
F	61	1	3	1.408476	112.8809	90.15067
F	61	1	3	1.401653	112.7761	-150.418
H	15	13	12	1.07974	121.5278	179.9066
H	55	52	49	1.091934	111.3828	-179.943

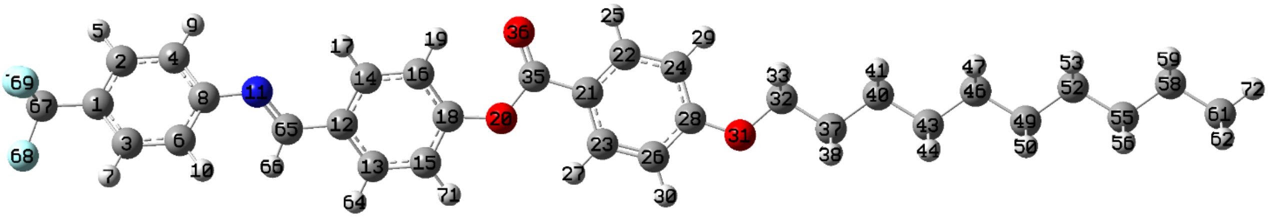

Symbol	NA	NB	NC	Bond	Angle	Dihedral
C						
C	1			1.402199		

C	1	2		1.399193	120.007	
C	2	1	3	1.388935	119.9597	0.820834
H	2	1	3	1.080183	119.8461	-179.498
C	3	1	2	1.391897	120.0143	-1.46468
H	3	1	2	1.080208	119.852	179.033
C	4	2	1	1.406036	120.5039	0.758222
H	4	2	1	1.079901	121.0731	-179.351
H	6	3	1	1.081023	119.8108	178.4817
N	8	4	2	1.409926	117.869	-179.112
C	11	8	4	2.416726	152.8332	-145.697
C	12	11	8	1.40725	146.3432	8.16133
C	12	11	8	1.406183	94.94318	-172.3
C	13	12	11	1.389813	120.6431	179.5653
C	14	12	11	1.392034	121.3485	-179.744
H	14	12	11	1.080289	118.4753	0.169172
C	15	13	12	1.397683	119.533	-0.12989
H	16	14	12	1.074863	120.9658	179.0778
O	18	15	13	1.410434	113.5645	178.4325
C	20	18	15	2.370683	161.2804	167.9888
C	21	20	18	1.403427	151.2641	-2.40679
C	21	20	18	1.409162	89.63985	178.3954
C	22	21	20	1.392113	120.9357	-179.019
H	22	21	20	1.080631	118.7008	0.978342
C	23	21	20	1.385843	120.3244	179.5539
H	23	21	20	1.078613	119.4446	-0.52498
C	24	22	21	1.402635	119.3673	-0.05223
H	24	22	21	1.078992	119.5561	179.984
H	26	23	21	1.079868	121.6107	179.9575
O	28	24	22	1.382586	124.3166	179.9975
C	31	28	24	1.466364	120.1528	-0.45936
H	32	31	28	1.09386	109.2751	59.7881
H	32	31	28	1.093882	109.2483	-59.2211
C	20	18	15	1.399088	126.095	167.385
O	35	20	18	1.234471	123.0516	-0.34601
C	32	31	28	1.520917	107.0211	-179.692
H	37	32	31	1.092791	108.6583	-58.4683
H	37	32	31	1.092821	108.5875	57.41245
C	37	32	31	1.53874	112.4437	179.4219
H	40	37	32	1.096225	109.6028	58.05924
H	40	37	32	1.096203	109.5462	-58.2291
C	40	37	32	1.538455	112.989	179.9414
H	43	40	37	1.095952	109.2004	-58.2211
H	43	40	37	1.095988	109.1833	57.55436
C	43	40	37	1.537945	113.3673	179.6451
H	46	43	40	1.096387	109.2597	57.86291
H	46	43	40	1.096376	109.2325	-57.9303

C	46	43	40	1.538263	113.4782	179.9819
H	49	46	43	1.096325	109.2254	-58.0408
H	49	46	43	1.09635	109.2149	57.70486
C	49	46	43	1.538228	113.4981	179.821
H	52	49	46	1.096451	109.2257	57.87088
H	52	49	46	1.096439	109.2129	-57.865
C	52	49	46	1.538031	113.551	-179.99
H	55	52	49	1.096534	109.2656	-57.9323
H	55	52	49	1.096551	109.2604	57.82922
C	55	52	49	1.538454	113.5724	179.9463
H	58	55	52	1.095564	109.1323	57.81999
H	58	55	52	1.095548	109.1309	-57.7911
C	58	55	52	1.535962	113.2318	-179.986
H	61	58	55	1.093028	111.1006	-59.84
H	61	58	55	1.093029	111.1035	59.94092
H	13	12	11	1.082883	119.7804	-0.44737
C	11	8	4	1.29	122.4066	-141.505
H	65	11	8	1.095893	121.2499	4.616855
C	1	3	6	1.485199	120.0231	-177.07
F	67	1	3	1.40147	112.815	-29.4503
F	67	1	3	1.408471	112.8813	90.13184
F	67	1	3	1.40165	112.7768	-150.436
H	15	13	12	1.07974	121.5276	179.9033
H	61	58	55	1.091996	111.4	-179.949

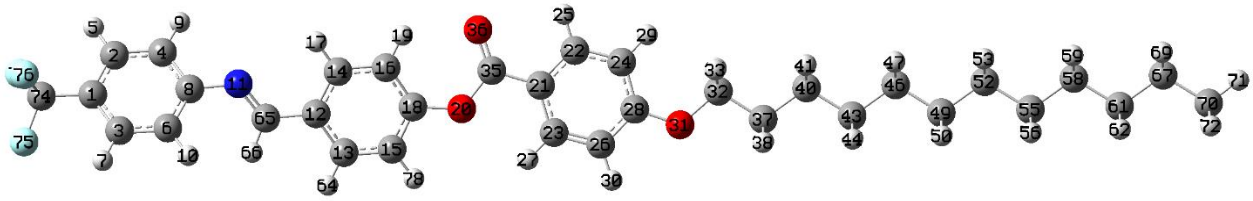

Symbol	NA	NB	NC	Bond	Angle	Dihedral
C						
C	1			1.402195		
C	1	2		1.399193	120.0075	
C	2	1	3	1.388941	119.9598	0.818629
H	2	1	3	1.080181	119.8467	-179.499
C	3	1	2	1.391899	120.0139	-1.46274
H	3	1	2	1.080207	119.8526	179.0344
C	4	2	1	1.406029	120.5033	0.757845
H	4	2	1	1.0799	121.0728	-179.349
H	6	3	1	1.081023	119.8115	178.4817

N	8	4	2	1.409932	117.8693	-179.105
C	11	8	4	2.416709	152.836	-145.707
C	12	11	8	1.407257	146.3451	8.175825
C	12	11	8	1.406176	94.94128	-172.28
C	13	12	11	1.38981	120.6428	179.5736
C	14	12	11	1.392035	121.3487	-179.75
H	14	12	11	1.08029	118.4755	0.165057
C	15	13	12	1.397685	119.5332	-0.12936
H	16	14	12	1.074863	120.966	179.0765
O	18	15	13	1.410428	113.5667	178.4214
C	20	18	15	2.370697	161.2752	168.0526
C	21	20	18	1.403431	151.2621	-2.4713
C	21	20	18	1.409166	89.64158	178.3089
C	22	21	20	1.39211	120.9361	-179.049
H	22	21	20	1.080631	118.7005	0.949384
C	23	21	20	1.385841	120.3237	179.5704
H	23	21	20	1.078613	119.4455	-0.5113
C	24	22	21	1.402641	119.3663	-0.05204
H	24	22	21	1.078995	119.5584	179.9831
H	26	23	21	1.079867	121.6109	179.9543
O	28	24	22	1.382585	124.3161	-179.995
C	31	28	24	1.466367	120.1511	-0.41423
H	32	31	28	1.093861	109.2737	59.74615
H	32	31	28	1.093882	109.2481	-59.2619
C	20	18	15	1.399089	126.0911	167.3807
O	35	20	18	1.234474	123.0502	-0.37977
C	32	31	28	1.520915	107.0222	-179.736
H	37	32	31	1.092792	108.6561	-58.4319
H	37	32	31	1.09282	108.5913	57.4497
C	37	32	31	1.538741	112.4423	179.4624
H	40	37	32	1.096224	109.601	58.05828
H	40	37	32	1.096204	109.5474	-58.2295
C	40	37	32	1.538455	112.9906	179.939
H	43	40	37	1.095953	109.2008	-58.2077
H	43	40	37	1.095986	109.1844	57.5687
C	43	40	37	1.537943	113.3653	179.6597
H	46	43	40	1.096388	109.259	57.85894
H	46	43	40	1.096379	109.2316	-57.9326
C	46	43	40	1.538256	113.4819	179.9787
H	49	46	43	1.096337	109.2263	-58.0498
H	49	46	43	1.096362	109.2148	57.69588
C	49	46	43	1.538198	113.494	179.8102
H	52	49	46	1.096431	109.23	57.86806
H	52	49	46	1.096423	109.2139	-57.8697
C	52	49	46	1.53826	113.5422	-179.991
H	55	52	49	1.096401	109.2181	-57.9512

H	55	52	49	1.096418	109.2101	57.77443
C	55	52	49	1.538245	113.5325	179.9048
H	58	55	52	1.096472	109.2177	57.87829
H	58	55	52	1.096462	109.2098	-57.8423
C	58	55	52	1.53804	113.5685	-179.978
H	61	58	55	1.096562	109.2618	-57.8975
H	61	58	55	1.096575	109.2575	57.85474
H	13	12	11	1.082884	119.7802	-0.44167
C	11	8	4	1.289994	122.409	-141.506
H	65	11	8	1.095892	121.2507	4.623593
C	61	58	55	1.538457	113.5867	179.9776
H	67	61	58	1.095558	109.1296	-57.7836
H	67	61	58	1.095571	109.1299	57.82177
C	67	61	58	1.535984	113.2386	-179.981
H	70	67	61	1.092031	111.4086	-179.954
H	70	67	61	1.093043	111.0968	-59.8399
H	70	67	61	1.093042	111.0993	59.93214
C	1	3	6	1.485199	120.0229	-177.067
F	74	1	3	1.401467	112.8155	-29.4585
F	74	1	3	1.408483	112.8812	90.12306
F	74	1	3	1.401653	112.7775	-150.445
H	15	13	12	1.079743	121.5279	179.899
